# Influence of Amine Compounds on the Thermal Stability of Paper-Oil Insulation

**DOI:** 10.3390/polym10080891

**Published:** 2018-08-09

**Authors:** Ningchuan Liang, Ruijin Liao, Min Xiang, Yang Mo, Yuan Yuan

**Affiliations:** 1 State Key Laboratory of Power Transmission Equipments & System Security and New Technology, School of Electrical Engineering, Chongqing University, Chongqing 400044, China; 20131102023@cqu.edu.cn (N.L.); rjliao@cqu.edu.cn (R.L.); xiangmin@cqu.edu.cn (M.X.); moyang_cqu@163.com (Y.M.); 2 College of Material Science and Engineering, Chongqing University, Chongqing 400044, China

**Keywords:** amine thermal stabilizer, thermal aging, ammonia, copper compound, front-line orbital energy

## Abstract

Amine compounds can greatly enhance the thermal stability of the insulating paper used in paper-oil insulation. Many research documents focus on paper‘s excellent thermal stability, but less attention has been paid to the effect of oil on paper’s degradation. In this research paper, we study the influence of different amine compounds on the thermal stability of both paper and oil, and a mechanism for the influence on paper-oil insulation as well as an optimal formula are proposed. First, six groups of paper were modified with different proportions of dicyandiamide (DICY), melamine, and polyacrylamide (PAM). Then, an accelerated thermal aging test at 130 °C was conducted for 30 days and the thermal aging characteristics of the oil-modified paper insulation were measured. The results showed that the thermal stability of the insulation paper modified with the amine compounds was remarkably improved, and P2 (2.25 wt % melamine, 0.75 wt % DICY, and 0.2 wt % PAM) presented the best anti-aging properties. However, certain properties of oil were influenced, such as acid value, and it was found that the ammonia produced by the amine stabilizers increased the copper compound content, which led to the deterioration of the insulating oil. Moreover, using a front-line orbital energy analysis by molecule modeling, it was determined that melamine was the core thermal stabilizer for the paper among the three amine compounds used in P2.

## 1. Introduction

The transformer is the core equipment in a power system. During operation, the transformer insulation system consisting of insulating paper and insulating oil is affected by temperature, electric field, moisture, oxygen, and other factors. The paper-oil insulation system gradually ages, causing the insulation performance to decrease, which in turn causes transformer insulation failure [1,2,3,4]. The aging of insulation paper in a paper-oil insulation system is irreversible, and its performance directly determines the service life of the transformer.

Kraft paper is a commonly used insulating paper for transformers, and its main component is natural cellulose [5]. Its low price, high initial mechanical strength, and electrical strength make it the preferred insulation material for most oil-immersed transformers [6,7,8]. However, cellulose is prone to thermal degradation, hydrolysis, and oxidative degradation during the transformer’s long-term operation [9,10,11,12]. The formation of small molecular substances such as small molecular acids and water leads to a decrease in the degree of polymerization (DP) and a decrease in the mechanical strength, which affect the transformer’s safe and stable operation [13,14,15]. Therefore, modifying the natural cellulose insulation paper and reducing the decrease rates of the DP and the mechanical strength of the insulation paper have become important research topics.

Two methods are available for modifying cellulose insulation paper: chemical and physical modification. Chemical modification replaces the hydroxyl group of the water-absorbing group in the cellulose by a more stable group (e.g., acetylation and cyanide ethylation) [16]. Physical modification refers to the addition of a thermal stabilizer to the insulating paper, and to the reaction of the thermal stabilizer with moisture, acid, and similar factors during aging, which slows the degradation rate of the insulating paper. The commonly used thermal stabilizers are amine compounds such as dicyandiamide (DICY), melamine, urea, polyacrylamide (PAM), and so on [17,18]. The insulating paper’s mechanical strength mainly depends on the strength of the cellulose chain itself and on the hydrogen bonds between the cellulose chains. After the chemical modification, the hydroxyl groups on the cellulose chains become fewer, which destroys the connection between the cellulose chains and causes a decrease in mechanical strength. In contrast, physical modification does not have this problem. Therefore, most of the thermal aging-resistant insulation papers used in transformers currently in operation are physically modified. Some scholars have studied DICY, melamine, and urea as amine compounds, and found that DICY-added insulation paper had a significant anti-aging effect, followed by melamine, and finally urea with the worst effect [18,19]. Moreover, melamine, DICY, and PAM were more effective than those that were added separately [20]. However, there are fewer explanations about the influence of amine compounds on paper-oil insulation.

In this research paper, six groups of insulating paper modified by DICY, melamine, and PAM were subjected to an oil immersion treatment, and an accelerated thermal aging test at 130 °C was performed. Then, the relevant thermal aging parameters were sampled and measured. By comparing the effect of different groups on the thermal stability of paper-oil insulation, the optimal formula for insulating paper was obtained, and the influence mechanism on the thermal stability of paper-oil insulation was studied.

## 2. Thermal Aging Test of Paper-Oil Insulation

### 2.1. Preparation of Thermal Aging Samples

**Figure 1:**
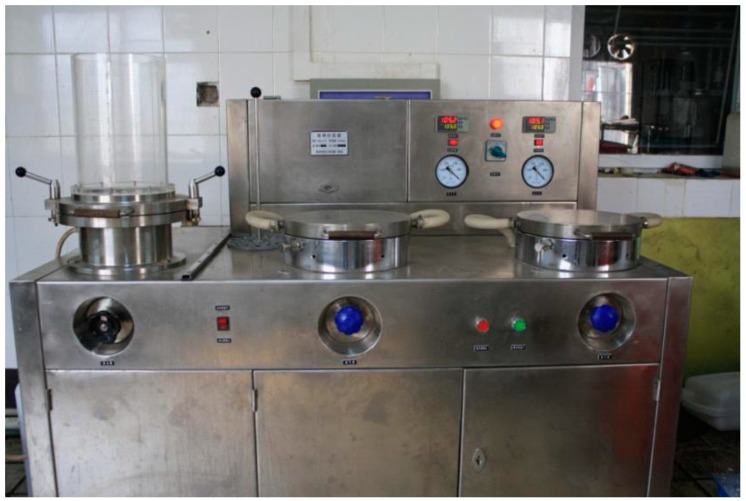
ZQJ1-B-II pattern shaper.

The test paper was made on the laboratory’s special beating and copying platform by a ZQJ1-B-II handsheet former (Annimat Instrument Co. Ltd., Jinan, Shandong, China), as shown in Figure 1. The raw material was pure sulfate wood pulp (Ilim Pulp Enterprise, Ustecky, Russia). DICY, melamine, and PAM (Sinopharm Chemical Reagent Co. Ltd., Shanghai, China) were selected as amine compounds.

PAM is a polymeric compound that contributes to flocculation during the papermaking process when over dose happened. Therefore, a small amount of PAM was added, and the addition amount remained fixed in the modified insulating paper. Because of the various types of amine stabilizers and their different nitrogen contents, a nitrogen content is commonly used to evaluate the stabilizer content in insulating paper, and the nitrogen content of most of the amine modified paper is between 1% and 4% [21]. Therefore, papers with different DICY and melamine contents and the same PAM content were made, and plain insulating papers without any thermal stabilizer were made as a reference. Six groups of samples were subjected to the thermal aging test. The addition of the amine compounds was done by coating.

### 2.2. Determination of the Amine Compound Thermal Stabilizer

The combination of DICY, melamine, and PAM has been found to have a better thermal stability than most of the amine stabilizers [22]. However, explanations about the influence of that combination on paper-oil insulation and about how the combination could be optimized are lacking. We investigated the influence mechanism of the combination of DICY, melamine, and PAM on the thermal stability of a paper-oil insulation system, and the best formula was obtained. The nitrogen content was considered as the main factor in improving the thermal stability of the insulating paper. Therefore, the total nitrogen content of the modified paper was set at 2 wt % according to the widely used nitrogen content of commercial thermally upgraded paper. Since the nitrogen content of PAM was smaller (16.7%) compared to DICY (66.7%) and melamine (66.7%), and the addition amount was limited by the insulating paper’s preparation process, the addition amount was fixed in each group. The effect of DICY, melamine, and PAM on the thermal stability of the insulation paper was studied under the premise that the total nitrogen content was constant. The nitrogen content was measured by a KDA-04A protein detector (Shanghai Beicheng Electric Equipment Factory, Shanghai, China). Six groups of samples were prepared shown in Table 1 as below:

### 2.3. Thermal Aging Properties of Paper and Oil

To study the effect of amine stabilizers on the thermal aging performance of paper-oil insulation, the thermal aging characteristics of the oil-impregnated insulating paper and oil were tested and analyzed using an accelerated thermal aging test. First, samples were dried at 50 Pa and 90 °C for 48 h. Then, the dried paper was impregnated with 25^#^ Xinjiang Karamay mineral insulating oil, which was degassed at 50 Pa and 40 °C for 24 h. The oil and paper were placed in a ground glass jar under nitrogen atmosphere, keeping the paper-oil ratio of 1:20. An appropriate amount of copper strip was added before the jar was sealed.

The sealed samples were placed in a 130 °C aging chamber for accelerated thermal aging. Parameters such as nitrogen content, degree of polymerization (DP) of cellulose, acid value, and dielectric loss of oil were measured, and the sampling time was 30 days. For convenience, the insulating papers modified with S0, S1, S2, S3, S4, and S5 were recorded as P0, P1, P2, P3, P4, and P5, and the insulating oils corresponding to the insulating paper were recorded as O0, O1, O2, O3, O4, and O5, respectively. Because of the low solubility of additives in oil and the low exchange speed between oil and paper, the initial values of oil tend to be similar. Hence, only 30-day samples of oil were sampled for analysis.

#### 2.3.1. Nitrogen Content

**Figure 2:**
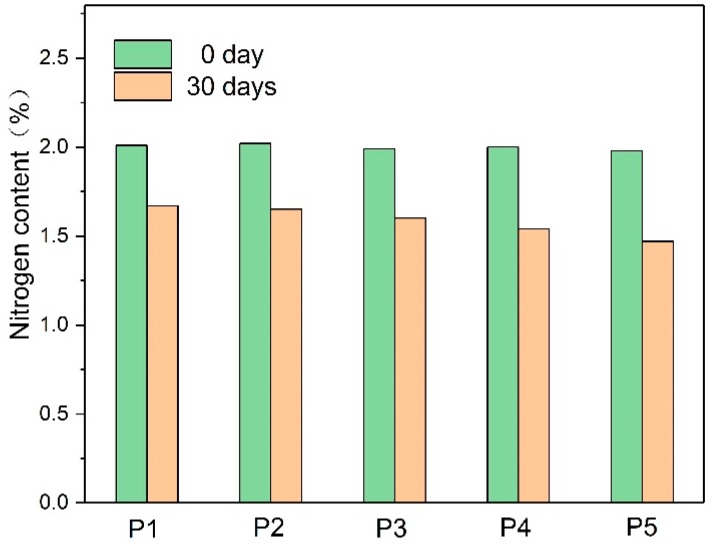
The nitrogen content of the different amine compounds.

The amine compound content in the insulating paper was characterized by the nitrogen content. During thermal aging, the amine compound content changed following the interaction of the thermal aging products of the paper. The nitrogen content in the insulating paper is shown in Figure 2.

During the 30-day aging process, the initial nitrogen content in the insulation paper showed a slight difference, and the nitrogen content in the insulation paper decreased after thermal aging. A small amount of the amine compounds diffused into the oil, whereas the insulation paper aging products (e.g., water and acid) consumed the amine compounds, thereby reducing the nitrogen content in the insulating paper. The nitrogen content of the insulating paper with P5 was reduced even more, reaching 27.4%. Papers containing less DICY showed a lower decrease of nitrogen content, whereas P1 obtained the least decrease.

#### 2.3.2. Tensile Strength and DP of the Insulating Paper

The tensile strength (i.e., the capability of a material or component to resist damage when pulled) can act as an indicator of paper’s mechanical performance. When tensile strength drops to 50%, the life of insulating paper is terminated. The DP is another commonly used parameter to evaluate the life of insulating paper. The DP refers to the number of glucose repeating units constituting the long chain of fibers in the insulating paper. An AT-L-1 tensile testing machine (Annimat Instrument Co. Ltd., Jinan, Shandong, China) was used to measure the tensile strength based on ISO 5270:1998, and an NCY-2 automatic viscometer (Srida Scientific Instrument Co. Ltd., Shanghai, China) was used to measure the DP based on ASTM D4243.

**Figure 3:**
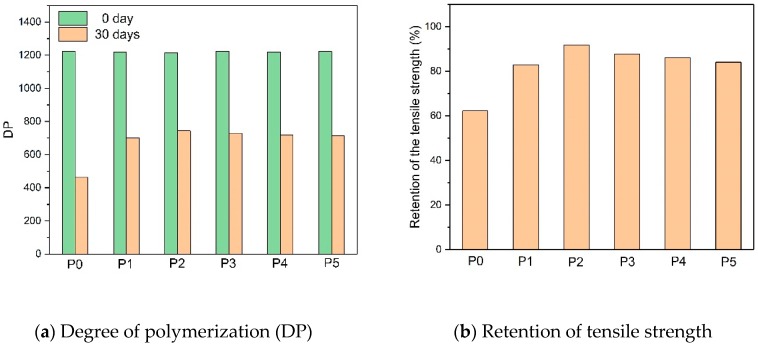
The influence of the different amine compounds on the thermal stability of paper.

Figure 3 shows that the initial DP of the six paper groups was around 1220 and there were fewer differences before aging. After the 30-day aging process, the DP of the modified insulating papers (P1–P5) were higher than the DP of P0. Moreover, the DPs of P1 and P5 were lower than those of the other modified insulating papers, indicating that DICY and melamine should be added together. In addition, P2 had the highest DP, reaching 751—much higher than the DP of P0, which reached 450. The retention of tensile strength is another way to evaluate the aging level of insulting paper, and the 50% tensile strength had been considered to be the end of useful life. The overall retention results agreed with the DP results, whereas P2 had a higher retention of tensile strength than other modified papers.

#### 2.3.3. Acidity in Oil

**Figure 4:**
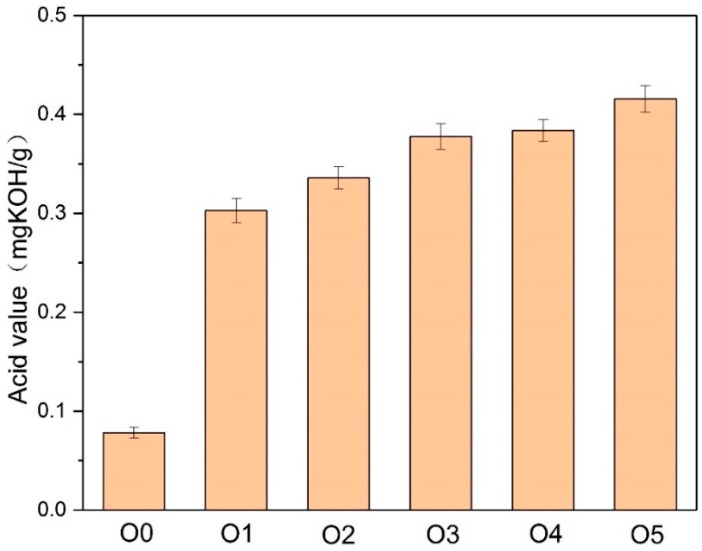
The influence of the different amine compounds in the paper-oil insulation on the acid value of oil after the aging process.

Among the acid substances produced by the thermal aging and decomposition of paper-oil insulation, small molecular acids are mainly produced by the aging decomposition of the insulating paper. The strong hydrophilicity is mainly adsorbed in the insulating paper, whereas the macromolecular acid is decomposed by the aging of the insulating oil. The distribution of the acid in the insulating oil can reflect the aging of the oil. Therefore, the acid content in the oil is important for measuring the aging of the insulating oil. This research paper used a Metrohm 907 Titrando automatic potentiometric titrator (Metrohm AG, Herisau, Switzerland) to measure the acid content in the oil in accordance with IEC 62021-1-2003. Figure 4 shows the measurement of the acid content in the oil after the aging test.

After the 30-day thermal aging, the acid value of the insulating oil with amine compounds was several times higher than that of O0. The amine compounds tended to enhance the acidity of the insulating oil during the thermal aging. In addition, the acid value of O1 was lower than that of O2–O5. Because of the absence of DICY in P1, the acid value was more likely to increase.

#### 2.3.4. Dielectric Loss Factor of Oil

The dielectric loss factor is an important indicator to reflect the quality of oil insulation properties. A DTL C dielectric instrument (BAUR GmbH, Sulz, Austria) was used to measure the dielectric properties of the immersed insulating paper and insulating oil. The test temperature was 90 °C, and the test frequency was 50 Hz, according to IEC 60156:1995.

**Figure 5:**
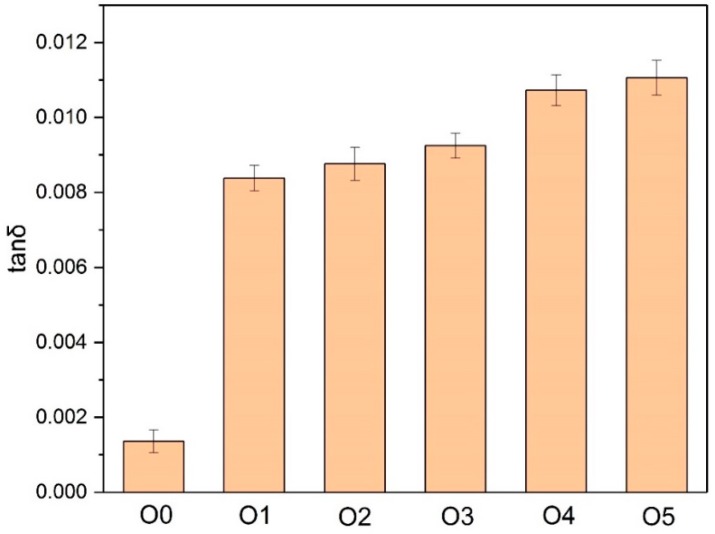
The influence of the different amine compounds on the dielectric loss of oil.

As shown in Figure 5, after the 30-day thermal aging, the dielectric loss factors of O1–O5 were higher than that of O1, indicating that under the same aging time, the aging products of O0 were smaller than in the corresponding insulating oils from S1–S5 with the added amine compounds. Among them, O5 got the largest tanδ, indicating that the most polarizing substances were produced in the aging process of O5, whereas the dielectric losses of O1 and O2 were relatively low, only higher than O0.

In general, it was obvious that paper P2 had the best thermal stability while having less of an impact on the oil.

## 3. Results and Discussion

Under the action of heat, water, acid, and oxygen, insulation paper will undergo three degradation reactions: pyrolysis, hydrolysis, and oxidation. Among them, the most critical factors to promote aging are water, acid, and temperature, whereas the effect of oxygen is not as obvious. There are three main types of water-soluble acids in transformer paper-oil insulation: formic acid, acetic acid, and levulinic acid. Water-soluble acids and water molecules form a synergistic effect to promote the cellulose hydrolysis reaction, thereby accelerating the degradation of the insulating paper. Amines inhibit cellulose degradation by depleting acids and water in paper-oil systems [23]. Most of amines are alkalescent, and can be neutralized with acids. The lone electron pairs on the amino nitrogen combine with hydrogen ions to form a covalent bond. Nitrogen changes from trivalent to ammonium salt:

(1) $$R-NH_{2}+H^{+}\to {\left[R-NH_{3}\right]}^{+}.$$

The reaction of DICY, melamine, and PAM with water is more complicated. DICY, melamine, and PAM can be hydrolyzed in an acid condition, and the main chemical reactions are as follows:

*DICY*:
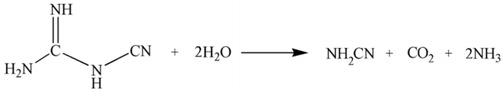
(2)

*Melamine*:
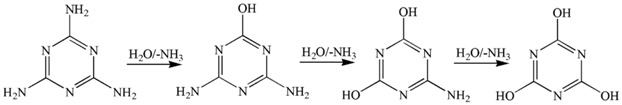
(3)

*PAM*:
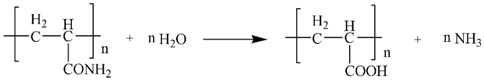
(4)

Since amine compounds consumed the water and the small molecular acid, the degradation of paper had obviously slowed down according to the DP and the retention of tensile strength. However, the reaction of amine compounds harmed the insulating properties of the insulating oils during the thermal aging.

Ammonia was produced during the thermal aging of the paper modified with DICY, melamine, or PAM. The reaction between ammonia and copper compounds is described as Equation (5) below:

(5) $$Cu^{2+}+4NH_{3}⇄{\left[Cu(NH_{3}{)}_{4}\right]}^{2+}.$$

Ammonia can form relatively stable complex ions with copper ions [24], which will change the standard electrode potential of copper ions and copper. According to the Nernst equation:

(6) $$E^{\theta }({\left[Cu{{(\mathrm{NH}}_{3})}_{4}\right]}^{2+}/Cu)=E^{\theta }(Cu^{2+}/Cu)+(0.0592/2)\mathrm{lg}[c(Cu^{2+})/c(Cu)].$$

According to the Nernst equation, the concentration of solid and liquid were considered as constant, defined as 1. The concentration of oxidant c(*Cu*^2+^) is quite a bit smaller than the concentration of reductant c(*Cu*) and it is obvious that:

(7) $$\mathrm{lg}[c(Cu^{2+})/c(Cu)]<0\Rightarrow (0.0592/2)\mathrm{lg}[c(Cu^{2+})/c(Cu)]<0.$$

(8) $$\mathrm{Hence},\;\;\;\;\;\;\;\;\;\;\;E^{\theta }({\left[Cu{{(\mathrm{NH}}_{3})}_{4}\right]}^{2+}/Cu)<E^{\theta }([Cu^{2+}/Cu).$$

Therefore, the potential of the standard electrode between the copper ion and the copper element is reduced, so that the elemental copper is more easily oxidized and causes the increasing copper compounds, which leads to the acceleration of the oil’s aging rate.

To further analyze the influence mechanism of amine compounds on the thermal stability of paper-oil insulation, inductively coupled plasma mass spectrometry (ICAP-QC, Thermo Fisher Scientific, Waltham, MA, USA) was used to quantitatively measure the copper content of the insulating paper and the insulating oil.

**Figure 6:**
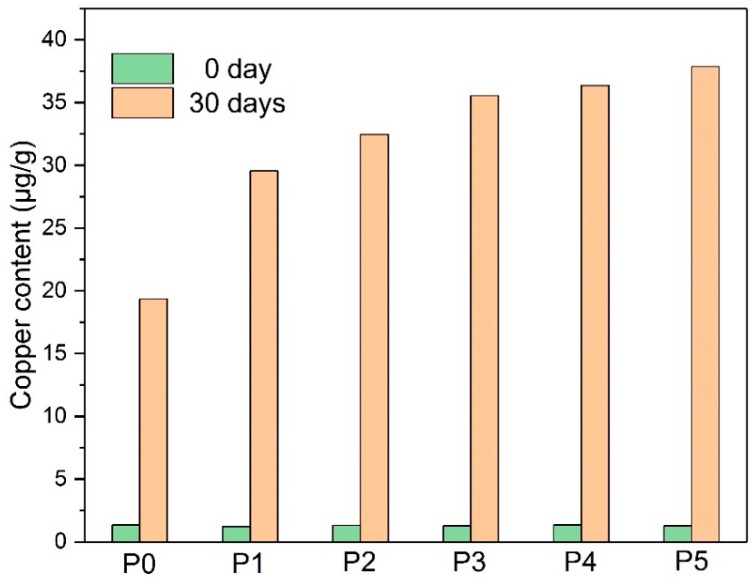
The variation of the copper content in the paper with the aging process.

As shown in Figure 6, there were fewer copper compounds in the paper at the start of the thermal aging. The paper adsorbed part of the copper compounds during the aging process, whereas P0 got the minimum copper content among the six groups. In comparison, the paper modified with amine stabilizers obtained a higher copper content, and the paper with more DICY absorbed much more copper compounds after the 30-day thermal aging.

**Figure 7:**
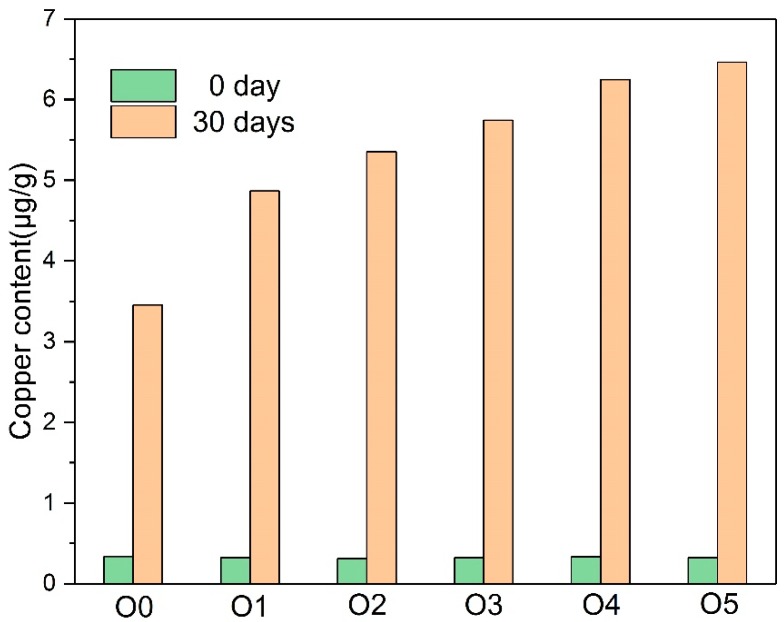
The variation of the copper content in the oil with the aging process.

In Figure 7, the copper content in the insulating oil was consistent with the copper content in the insulating paper. The copper compound in the oil was at a very low level at the beginning of the thermal aging. The copper content of the oil increased significantly after the 30-day thermal aging, and the copper contents of the oils with the amine compounds (O1–O5) were higher than the oil with plain paper (O0). O1 without melamine had fewer copper compounds compared to other oils with modified paper.

Considering the different contents present in the six sets, the chemical reactions between the components of amine stabilizers and the factors accelerating thermal aging (water and small molecular acid) were investigated. In the intermolecular chemical reactions, the intermolecular frontier orbital energy gap determined the chemical reaction activity [25]. The smaller the energy gap, the easier the reaction, and the larger and the more stable it was. By analyzing the front-line orbital energy gap using density-functional calculations of related molecules through Materials Studio 4.0, the chemical reaction activity between modified paper (DICY, melamine, PAM, β-d-glucopyranose (unit of cellulose)) and the thermal aging acceleration factors (water, formic acid, acetic acid, and levulinic acid) were examined, as shown in Table 2.

Considering the limited addition amount of PAM, PAM tended to function well at the beginning of the thermal aging. In fact, it obtained the lowest energy gap between the water and the small molecular acid. DICY reacted much more easily with water compared to melamine, but the energy gap between the small-molecule acid was close to the β-d-glucopyranose. Hence, DICY tended to react with water, and more ammonia was produced according to Equation (2), which accelerated the aging speed of the oil. Compared with DICY, the energy gap between both the water and the small molecular acid was obviously smaller than with β-d-glucopyranose, which means melamine consumed both the water and the small molecular acid during the entire thermal aging process.

The moisture content measurement of the insulating paper in this experiment was performed by a DL32D Coulometric Karl Fischer titrator and a DO308 drying oven (Metrohm Co. Ltd., Herisau, Switzerland).

**Figure 8:**
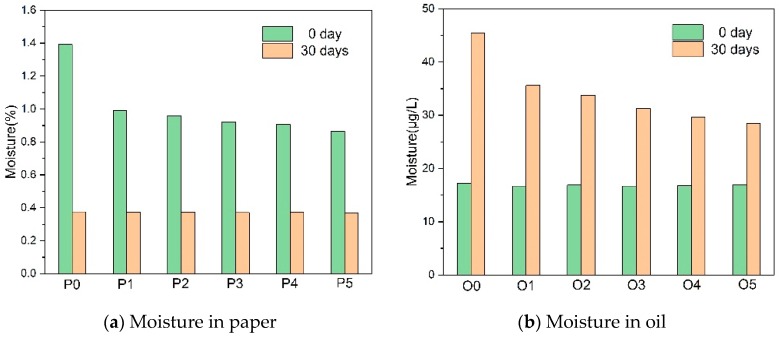
The variation of the moisture in the paper and the oil with the thermal aging.

Figure 8 shows the moisture in both paper and oil. The amine compounds depleted the moisture in the paper, which led to the decrease of moisture in the oil, because of the diffusion and high moisture in the paper produced by the cellulose degradation. Compared with melamine, DICY reacted much more easily with water, and the oil in the groups with DICY (O2–O5) had lower moisture, whether in the paper or in the oil.

In fact, water affects the concentration of H^+^ ions by hydrolyzing the acid, whereas the hydrolysis reaction of paper mostly depends on the H^+^ ion concentration. Because of the 0.5 wt % initial moisture and the rare small-molecule acid in the paper before aging, the H^+^ ion concentration was restricted by the small-molecule acid. It was more effective to control the amount of the small molecular acid than the moisture. As the thermal aging progressing, the water tended to be another restriction factor of acid hydrolysis as more and more small molecular acid has been produced by the cellulose degradation. The large contents of melamine and PAM (P1) consumed the water and the small molecular acid, which greatly alleviated the thermal degradation of the paper. To further enhance the thermal stability, small content of the DICY(P2) was added to consuming more water. However, because of the constant nitrogen content, the excessive addition of the DICY reduced the melamine content. This led to the decrease of the thermal stability of the paper (P3–P5) because the melamine was more reactive with the small molecular acid. Moreover, the great quantity of ammonia produced by the DICY accelerated the deterioration of the oil. The aging products of the insulating oil also contained oxidants, which accelerated the aging of the insulating paper and, therefore, could not be ignored [26].

## 4. Conclusions

This work investigated the thermal aging characteristics of modified insulating paper and general insulating paper with oil at 130 °C. The nitrogen content, DP, tensile strength, copper content, and moisture of the insulating paper were measured, and various parameters, such as acid value, dielectric loss, copper content, and moisture in the oil were compared and analyzed to arrive at the following conclusions:

- The papers modified with melamine, DICY, and PAM (P2, P3, and P4) had better thermal stability, whereas the addition of an amine thermal stabilizer accelerated the thermal aging of the insulating oil. P2 (2.25 wt % melamine, 0.75 wt % DICY, and 0.2 wt % PAM) obtained the best thermal stability and had less impact on the insulating oil during thermal aging.
- The addition of the amine compounds inhibited cellulose degradation by depleting the acids and the water in the paper-oil systems while producing ammonia, which led to the increase of copper compounds in the insulating oil, thereby increasing the aging rate of the insulating oil.
- Melamine was the core thermal stabilizer among the three amine compounds in P2 due to its lower energy gap between both the water and the small molecular acid and less defects than PAM and DICY.

## Figures and Tables

**Table 1:** The sample content. DICY: dicyandiamide, PAM: polyacrylamide.

Group	Content
S0	--
S1	3 wt % melamine + 0.2 wt % PAM
S2	2.25 wt % melamine + 0.75 wt % DICY + 0.2 wt % PAM
S3	1.5 wt % melamine + 1.5 wt % DICY + 0.2 wt % PAM
S4	0.75 wt % melamine + 2.25 wt % DICY + 0.2 wt % PAM
S5	3 wt % DICY + 0.2 wt % PAM

**Table 2:** Reactant molecules (eV).

	E_Gap_			
	β-d-glucopyranose	DICY	Melamine	PAM
Water	6.68584	6.13073	6.21782	5.90284
Formic acid	4.88445	4.74839	4.43546	4.12607
Acetic acid	4.57424	4.43818	4.12525	3.81104
Levulinic acid	4.28852	4.15246	3.83953	3.52481
